# Individual Differences in Reward Sensitivity Modulate the Distinctive Effects of Conscious and Unconscious Rewards on Executive Performance

**DOI:** 10.3389/fpsyg.2018.00148

**Published:** 2018-02-16

**Authors:** Rémi L. Capa, Cédric A. Bouquet

**Affiliations:** ^1^French National Institute for Health and Medical Research, Department of Psychiatry, University of Strasbourg, Strasbourg, France; ^2^L’institut National Universitaire Jean-François Champollion, Université de Toulouse, Albi, France; ^3^Centre National de la Recherche Scientifique, Centre de Recherches sur la Cognition et L’apprentissage, Université de Poitiers, Poitiers, France

**Keywords:** reward, conscious and unconscious processes, behavioral activation, individual differences, executive control, memory-updating

## Abstract

Executive control can be driven by conscious and unconscious monetary cues. This has raised the exciting question regarding the role of conscious and unconscious reward in the regulation of executive control. Similarities and differences have been uncovered between unconscious and conscious processing of monetary rewards. In the present study, we explored whether individual differences associated with reward sensitivity foster these variations on memory-updating—a core component process of executive control. Participants (*N* = 60) with low, medium, and high reward sensitivity were selected and performed a numerical memory-updating task. At the beginning of each trial, a high (1 euro) or a low (5 cents) reward was presented subliminally (24 ms) or supraliminally (300 ms). Participants earned the reward by responding correctly. Participants with low reward sensitivity performed better for the high reward only in the subliminal condition. For participants with medium reward sensitivity, performance improved with high reward in both subliminal and supraliminal conditions. When participants had high reward sensitivity scores, the effect of reward was stronger in the supraliminal condition than the subliminal condition. These results show that the distinctive effects of conscious and unconscious rewards on executive performance are modulated by individual differences in reward sensitivity. We discuss this finding with reference to models of conscious/unconscious processing of reward stimuli.

## Introduction

Executive control has been defined as “the ability to flexibly and dynamically adjust one’s performance to changing environmental demands and internal goal states” (Barch et al., 2009). This ability has been strongly linked to consciousness. However, several studies have reported effects of subliminal stimuli on high-order executive control processes (van Gaal et al., 2012). For instance, studies have suggested that conscious but also unconscious processing of monetary reward can increase performance in tasks requiring executive control (Capa et al., 2011, 2013; Bustin et al., 2012). This has raised the exciting question of the role of conscious and unconscious processing reward in executive control. However, differences have been uncovered between conscious and unconscious reward. In the present study, we explored whether the behavioral activation system – a motivational system responsible for organizing and regulating behavior to attain rewards (Gray, 1989) – may be a crucial factor to explain differences in executive performance (memory-updating) between conscious and unconscious reward processing.

Initial empirical evidence of the influence of unconscious reward processing was provided by Pessiglione et al. (2007), who invited participants to perform a task in which they could earn money by squeezing a handgrip. Participants put in more effort for larger sums of money displayed subliminally and supraliminally. Moreover, the same basal forebrain region was involved in both subliminal and supraliminal rewards presentation, which suggests that the cerebral structures involved in both conditions were qualitatively similar. This influential study was replicated and extended to various cognitive tasks (Bijleveld et al., 2009, 2010, 2011, 2012a; Capa et al., 2011, 2013; Zedelius et al., 2011, 2012a,b, 2013, 2014; Bustin et al., 2012). Among these studies, several showed that even performance in complex tasks involving high-order processes, traditionally thought to require consciousness, can be driven by both conscious and unconscious rewards. For instance, Bijleveld et al. (2010) invited participants to perform a task in which they could earn money by quickly and accurately solving a mathematical equation. The amount of money that participants received was contingent on their speed and accuracy. The possibility of speed-accuracy trade-off thus allowed participants to make strategic choices. In other words, participants could choose between using a rapid strategy or a cautious one. Subliminal high rewards made participants more eager, with faster but equally accurate responses. Supraliminal high rewards, on the other hand, caused participants to be more cautious, with slower but more accurate responses. Interestingly, other studies also reported differences between conscious and unconscious rewards for tasks requiring executive control such as memory-updating (Capa et al., 2011; Bustin et al., 2012) and task-switching (Capa et al., 2013).

In a previous study (Capa et al., 2011), we sought to investigate the influence of conscious and unconscious rewards on memory-updating. Participants had to memorize five numbers and update those numbers independently according to a series of six successive arithmetic operations. At the beginning of each trial, a reward (1 euro or 5 cents) was presented either subliminally (27 ms) or supraliminally (300 ms). If participants successfully reported the final correct series of numbers, then they earned the reward at stake. Results showed better performance when a high monetary reward (either consciously or unconsciously processed) was at stake. However, the participants showed a better percentage of correct responses when subliminal reward cues were presented compared to supraliminal cues.

In another study, we tested the influence of conscious and unconscious rewards during cued task-switching performance (Capa et al., 2013). In this study, participants performed runs of task-switching. During each run, participants switched among three tasks and earned the reward contingent upon their accuracy in the run. The percentage of correct runs was larger for the higher than for the lower reward, in both subliminal and supraliminal conditions. In respect to reaction times, participants were overall faster for the supraliminal reward. Moreover, for the subliminal reward, no reaction time difference was observed between high and low reward conditions suggesting that participants were more cautious.

Why consciously and unconsciously processed rewards can differentially affect executive control is open to argument and the modulating factors of the reward-related effects remain to be fully understood. In this context, one important – but rather unexplored – question is whether individuals’ personality traits or tendencies can modulate these effects on executive performance. The impact of individual differences on the distinctive effects of conscious and unconscious rewards is a key issue to investigate to further our understanding of the regulating role of motivation in executive control (Braver et al., 2010).

In a first attempt (Bustin et al., 2012), we explored whether individual differences associated with novelty seeking could foster differences in the effect of reward processing on executive function. Novelty seeking is defined as a trait involving activation or initiation of behaviors such as exploratory activity and approach to monetary rewards (Cloninger et al., 1993). Within this frame, participants performed a memory-updating task, similar to Capa et al. (2011), to earn rewards presented consciously and unconsciously. On the basis of participants’ scores on the novelty seeking scale from the Temperament and Character Inventory-Revised (TCI-R; Cloninger, 1999), two groups (low below the median vs. high above the median) of participants were created. We found that low novelty seeking participants performed better when rewards were presented subliminally, whereas high novelty seeking participants’ performance did not differ regardless whether reward cues were processed consciously or unconsciously. These previous findings highlight the necessity of taking individual differences into account to better understand the effects of conscious and unconscious processing reward on executive control.

To examine further this issue, the present study focused on reward sensitivity, which is known to be a moderator of reward processing (Kim et al., 2015). More specifically, we investigated whether individual differences in reward sensitivity can foster the distinctive effects of conscious (supraliminal) and. unconscious (subliminal) processing of reward on executive performance. Cloninger’s model of personality, which incorporates novelty seeking tendency (see above), is theoretically related to Gray (1989) model, which distinguishes between two motivational systems: the behavioral approach system (BAS) and the behavioral inhibition system (BIS) (Mardaga and Hansenne, 2007). Hence, the present work was guided by Gray’s model. Accordingly, the BIS guides’ behavior in response to punishment signals via the septohippocampal system. The BAS, on the other hand, may organize and regulate behavior in response to reward signals via the dopamine system.

Reward sensitivity is measured using the BIS/BAS scale developed by Carver and White (1994). The BAS scale consists of three subdimensions, with two subscales related to reward processing (BAS Drive and BAS Reward Responsiveness) and one to novelty-seeking (BAS Fun Seeking). Previous research has shown that the BAS Drive and BAS Reward Responsiveness subscales can be used as a reliable index of individual differences in reward sensitivity (Carver and White, 1994; Hickey et al., 2010). The BAS Reward Responsiveness subscale captures positive responses to the occurrence or anticipation of reward, whereas the BAS Drive subscale indexes the persistent pursuit of desired rewards. Participants with high, as opposed to low, BAS Drive scores show correspondingly higher task engagement to earn a conscious reward during a cognitive task (Boksem et al., 2006, 2008; Hickey et al., 2010). Similarly, BAS Reward Responsiveness has been found to correlate positively with reward-related effects on cognitive processing (Braem et al., 2012). Differences in BAS scores can thus lead to differential effects of conscious rewards. To the best of our knowledge, no studies to date have looked at the impact of unconscious reward processing as a function of the BAS system.

In the present study, executive control was probed through a memory-updating task. Memory-updating is a key component of executive control. It refers to a process that is required to modify the content of working memory by replacing current, no longer relevant information with more relevant information (Morris and Jones, 1990). Participants were selected with low, medium, or high scores on the BAS Drive subscale.^1^ They could earn money displayed subliminally or supraliminally by performing well in a numerical memory-updating task. As observed previously in a similar task (Capa et al., 2011; Bustin et al., 2012), we anticipated a reward effect, with better performance associated with the possibility of earning a high reward as opposed to a low reward. Moreover, we explored whether conscious and unconscious reward processing differed or not as a function of the BAS system. We expected an increase in the reward effect on executive performance concomitant with the increase in BAS scores.

## Materials and Methods

### Participants

The French version of the BIS/BAS scales (Carver and White, 1994) was administered to 215 university students (129 female, 86 male) enrolled in an introductory-level psychology course. Their mean age was 21.18 years (*SD* = 3.13). Participants were classified as low BAS if their score (i.e., the sum of the four items) with the BAS Drive subscale was six or less (below the 15^th^ percentile), and high BAS if their score was 10 or more (above the 85^th^ percentile).^2^ Participants who scored 7 or 8 (40^th^ and 60^th^ percentile) were classified as medium BAS (participants with a score of 9 were excluded). Furthermore, participants in the low, medium, and high BAS groups were also selected if their BIS score (i.e., sum of the 7 BIS items) was between 14 and 22 (15^th^ and 85^th^ percentile). This was done to avoid differences in BIS scores across groups. Participants among this first sample of those who volunteered to take part in the study had to fill out the BIS/BAS questionnaire a second time (Carver and White, 1994). Only participants whose scores stayed within the limits set by the initial distribution were retained, as an experimental precaution to ensure their characteristics were stable. Therefore, a total of 60 participants (20 per group) constituted our final sample.

The experiment was conducted in accordance with the Helsinki Declaration. Each participant read and signed an informed consent form prior to taking part in the experiment. They were allowed to keep the money they earned. The study was of 2 (reward presentation duration: supraliminal vs. subliminal) × 2 (reward value: low vs. high) × 3 (BAS group: low vs. medium vs. high) mixed-factorial design. Reward presentation duration and reward value were within-participants factors, and BAS group was a between-participant factor.

### Experimental Task

**FIGURE 1:**
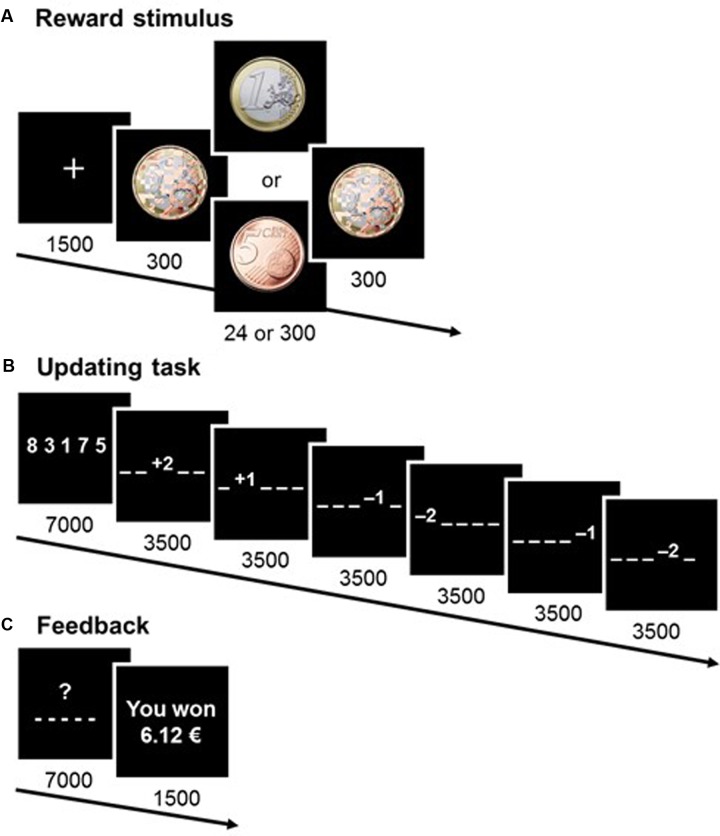
Design of the experimental task. A series of screens were displayed during a trial, with durations in milliseconds. At the beginning of each trial **(A)**, a reward of either 1 euro or 5 cents was shown on the screen, either subliminally (24 ms) or supraliminally (300 ms). To earn the reward, participants had to find the correct response in the updating task **(B)**. Finally, at the end of each trial **(C)** participants were informed of their cumulative earnings.

The updating task – based on the memory-updating paradigm devised by Salthouse et al. (1991) – was presented on a 85-Hz CRT screen. Participants took part in a training session consisting of eight trials (two trials per condition), followed by 80 experimental trials (20 repetitions per condition). At the beginning of each trial, a fixation cross appeared, followed immediately by a pre-mask, the reward stimulus (presented subliminally for 24 ms^3^ or supraliminally for 300 ms), and a post-mask (**Figure 1A**). Stimuli for the updating task were then presented (**Figure 1B**). Participants were instructed that if they responded correctly, they would receive the reward presented at the beginning of the trial. They had to memorize five numbers and update each number independently according to a series of six arithmetical operations (i.e., additions and subtractions of ±1 or ±2). Intermediate and end results for each number were always in the range of 0 to 9, to ensure a constant degree of difficulty.

At the end of the trial, participants were asked to enter the final value of each number on a keyboard. They were told they would only win the pecuniary reward if all five numbers were correct. The five-number sequences they had to memorize, the six successive updating operations, and the required responses were all different across trials. This ensured there was no implicit learning or association possible between the reward stimuli and the response. Cumulative earnings were displayed at the end of each trial (**Figure 1C**). Participants were told that the reward stimuli were either 1 euro or 5 cents and would sometimes be difficult to see. This was an experimental precaution which ensured that participants paid attention to the rewards. It was used because the cognitive processes at work in masked priming experiments are dependent on attention (Naccache et al., 2002).

### Perceptual Discrimination Task

To ensure that supraliminal reward stimuli were consciously perceived and subliminal reward stimuli were not, after the experimental task participants were asked to perform a forced-choice test. The test consisted of four training trials followed by 80 experimental trials. Each trial consisted of masks and reward cues (**Figure 1A**), after which several choices were displayed simultaneously instead of the experimental task. Participants were asked to choose one of four responses: “I saw 1 euro,” “I saw 5 cents,” “I guess it was 1 euro,” “I guess it was 5 cents.” There was no limit to the response time, and the possible responses remained on the screen until a choice had been made.

### Self-Report Data

After the perceptual discrimination task, participants filled out the French version of the BIS/BAS scales (Carver and White, 1994). The BIS scale consisted of seven items (e.g., “I feel worried when I think I have done poorly”). The BAS scale, for its part, was made up of three subscales: Drive (four items, “I go out of my way to get things I want”), Reward Responsiveness (five items, “When I get something I want, I feel excited and energized”), and Fun Seeking (four items, “I crave excitement and new sensations”). Participants rated their responses on four-point scales ranging from 1 (totally true) to 4 (totally wrong). In the current study, Cronbach’s alphas were 0.70 for the BIS scale, 0.71 for Drive, 0.73 for Reward Responsiveness, and 0.69 for Fun Seeking. Correlations between the BAS subscales ranged from 0.42 to 0.62. The BIS scale was unrelated to the BAS Reward Responsiveness and BAS Drive subscales but had a small negative association with the BAS Fun Seeking subscale (*r* = -0.21).

## Results

### Participant Characteristics

As expected, Student’s *t*-tests revealed that participants in the medium BAS group had higher BAS Drive scores than participants in the low BAS group and lower scores than participants in the high BAS group [*t*(38) = 12.52, *p* < 0.0001, *d* = 3.96] and [*t*(38) = 21.39, *p* < 0.0001, *d* = 6.79], respectively. Student’s *t*-tests revealed that participants with medium BAS had higher BAS Reward Responsiveness [*t*(38) = 3.47, *p* < 0.001, *d* = 1.09], BAS Fun Seeking [*t*(38) = 2.25, *p* < 0.03, *d* = 0.71], BAS Total [*t*(38) = 3.29, *p* < 0.002, *d* = 1.04] scores than participants in the low BAS group and also lower BAS Reward Responsiveness [*t*(38) = 2.80, *p* < 0.007, *d* = 0.89], BAS Fun Seeking [*t*(38) = 2.61, *p* < 0.01, *d* = 0.82], BAS Total [*t*(38) = 3.20, *p* < 0.003, *d* = 1.01] scores than participants in the high BAS group. Analyses of BIS scores revealed no significant difference between groups (all *p*s > 0.77). These results reflect a successful selection of participants. Their characteristics are presented in **Table 1**.

**Table 1:** Participants’ characteristics.

	Low BAS	Medium BAS	High BAS
Gender (F/M)	11/9	11/9	11/9
Age (M ± *SD*)	21.27 ± 2.05	21.38 ± 1.52	21.35 ± 1.48
BAS drive (M ± *SD*)	5.05 ± 0.76	7.60 ± 0.50	11.05 ± 1.00
BAS reward responsiveness (M ± *SD*)	7.80 ± 2.84	11.65 ± 4.07	14.05 ± 4.15
BAS fun seeking (M ± *SD*)	8.25 ± 2.36	9.85 ± 2.13	11.60 ± 2.11
BAS total (M ± *SD*)	21.10 ± 4.76	29.10 ± 5.49	37.80 ± 4.74
BIS (M ± *SD*)	16.80 ± 0.73	16.65 ± 1.25	16.75 ± 1.11


**BAS, behavioral activation system; BIS, behavioral inhibition system*.*

### Percentage of Correct Responses in the Updating Task

**FIGURE 2:**
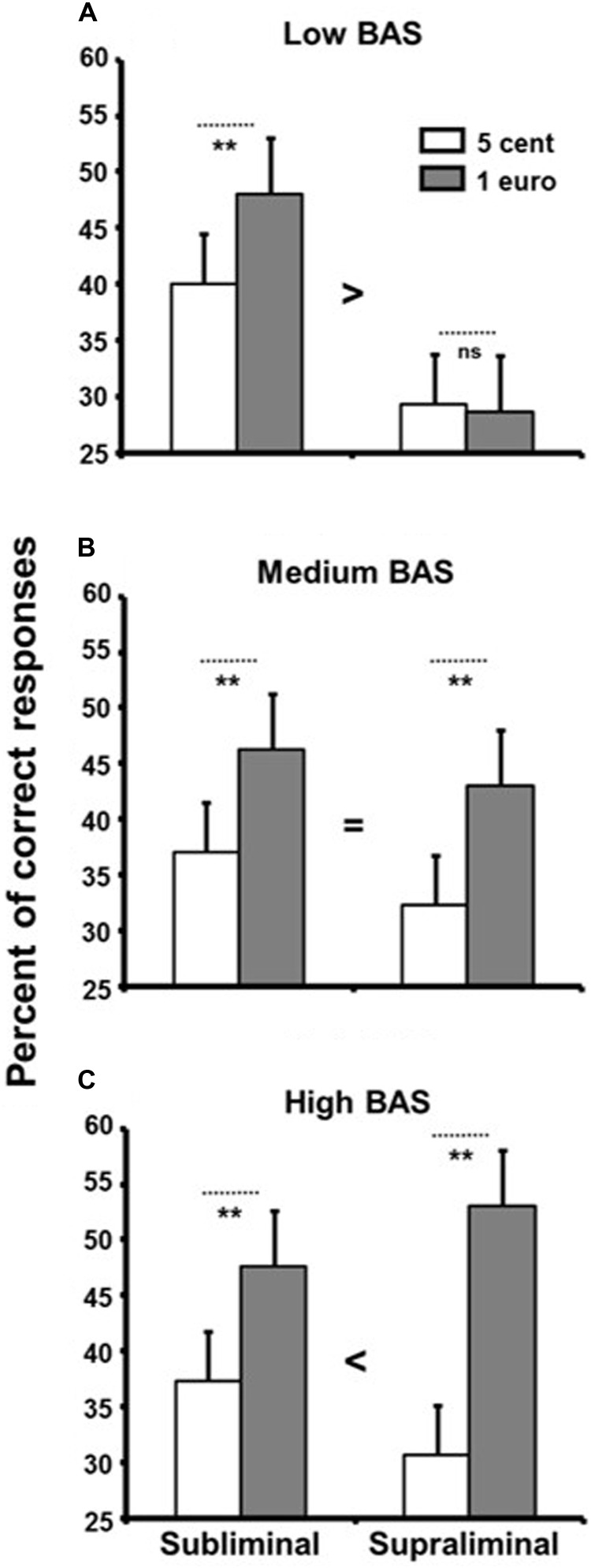
Percentage of correct responses in the memory-updating task as a function of reward value displayed subliminally and supraliminally and BAS groups. Low BAS participants **(A)** performed better for the high reward than the low reward only in the subliminal condition. In the medium BAS group **(B)**, there was a main effect of reward, and it was similar in the subliminal and supraliminal conditions. In the high BAS group **(C)**, the reward effect was higher in the supraliminal condition than the subliminal condition. Error bars denote standard errors of the mean. ^∗∗^*p* < 0.005, and ns for not significant.

A three-way ANOVA with reward presentation duration (subliminal vs. supraliminal) and reward value (1 euro vs. 5 cents) as within-participants factors and BAS group (low vs. medium vs. high) as between-participants factor was used to analyze the data. Reward value had a main effect, with better performance for the high reward (*M* = 44.00, *SD* = 24.79) than the low reward (*M* = 34.00, *SD* = 20.98), *F*(1,57) = 64.11, *p* < 0.0001, $\eta_{p}^{2}$ = 0.53, reflecting a generally successful manipulation of reward. Most interestingly, we found a significant interaction between reward presentation duration, reward value, and group, *F*(2,57) = 8.64, *p* < 0.005, $\eta_{p}^{2}$ = 0.23 (**Figure 2**).

We broke this interaction down by performing three separate ANOVAs (2 reward presentation duration × 2 reward value) for each BAS group. In the low BAS group (**Figure 2A**), there was a main effect of reward presentation duration, *F*(1,19) = 9.42, *p* < 0.007, $\eta_{p}^{2}$ = 0.33, with better performance in the subliminal condition (*M* = 44.00, *SD* = 26.57) than the supraliminal condition (*M* = 29.00, *SD* = 13.80). No main effect of reward value was found (*p* > 0.12), but there was a significant interaction between reward value and reward presentation duration, *F*(1,19) = 6.41, *p* < 0.02, $\eta_{p}^{2}$ = 0.25. The low BAS participants only performed better for the high reward than the low reward in the subliminal condition. Complementary Student’s *t*-tests confirmed that the reward effect was present only in the subliminal condition, *t*(19) = 3.21, *p* < 0.005, *d* = 0.70, and not in the supraliminal condition (*p* = 0.83).

In the medium BAS group (**Figure 2B**), there was a main effect of reward with better performance when a high reward was at stake (*M* = 44.67, *SD* = 22.86) as opposed to a low reward (*M* = 34.17, *SD* = 18.81), *F*(1,19) = 58.41, *p* < 0.0001, $\eta_{p}^{2}$ = 0.75. This effect was not affected by reward presentation duration (*p* = 0.57). To ascertain whether there was a reward effect in both conditions, complementary Student’s *t*-tests were conducted. Participants in the medium BAS group performed better for the high reward than the low reward, both in the subliminal condition, *t*(19) = 3.62, *p* < 0.002, *d* = 0.80, and the supraliminal condition, *t*(19) = 5.16, *p* < 0.0001, *d* = 1.11. The main effect of reward presentation duration was not significant (*p* = 0.21).

In the high BAS group (**Figure 2C**), the main effect of reward was significant (1 euro: *M* = 49.00, *SD* = 26.65, and 5 cents: *M* = 33.17, *SD* = 23.80), *F*(1,19) = 35.46, *p* < 0.0001, $\eta_{p}^{2}$ = 0.65. Furthermore, the reward effect was higher in the supraliminal condition than the subliminal condition, as suggested by a significant interaction, *F*(1,19) = 10.70, *p* < 0.004, $\eta_{p}^{2}$ = 0.36. Complementary Student’s *t*-tests showed an effect of reward in both conditions of presentation duration, *t*(19) = 3.32, *p* < 0.004, *d* = 0.75 for the subliminal condition and *t*(19) = 5.44, *p* < 0.0001, *d* = 1.13 for the supraliminal condition. No difference in performance was found between the possibility of earning 5 cents depending on whether it was displayed subliminally or supraliminally (*p* = 0.40). However, high BAS participants performed better in the subliminal condition in order to win 1 euro than in the supraliminal condition, *t*(19) = 2.21, *p* < 0.04, *d* = 0.47.

### Complementary Analysis

To gain a better understanding of the effect of individual differences associated with reward sensitivity for conscious and unconscious reward processes, we conducted two separate ANOVAs (2 reward value × 3 group), one for the subliminal and the other for the supraliminal condition. In the subliminal condition, no main effect of group or interaction was found (all *p*s > 0.81). In the supraliminal condition, the effect of reward increased with the increase in the BAS scores, as suggested by a significant interaction between reward value and groups, *F*(2,57) = 12.98, *p* < 0.001, $\eta_{p}^{2}$ = 0.31. Complementary Student’s *t*-tests showed that the reward effect (difference between 1 euro and 5 cents) in the medium BAS group (*M* = 11.67, *SD* = 10.12) was greater than that in the low BAS group (*M* = -0.67, *SD* = 14.00), *t*(38) = 3.19, *p* < 0.003, *d* = 0.62, but less than that in the high BAS group (*M* = 23.33, *SD* = 19.16), *t*(38) = 2.41, *p* < 0.02, *d* = 0.71.

### Prime Visibility Test

It was apparent from debriefing participants before the prime visibility test that none of them was able to report whether 1 euro or 5 cents coins were presented subliminally. We analyzed the prime visibility test results on the basis of correct responses, defined as responses indicating the participant had seen or guessed the right coin. Results of the prime visibility test showed that the participants had seen the coins in the supraliminal condition (*M* = 97.85). However, for the subliminal coins, the mean percentage of correct responses (*M* = 50.94, *SD* = 6.29) was not significantly different from chance, as suggested by Student’s *t*-tests (*p*s > 0.24). In addition, Student’s *t*-tests revealed no difference between groups (*p*s > 0.72).

## Discussion

The present study shows that executive performance associated with the possibility of earning a high reward improved with the increase in the BAS scores when the reward was consciously processed, but not when the reward was subliminally displayed. In the same way that personality has been shown not only to attenuate, but even sometimes to eradicate or reverse classic psychological effects (Matthews, 2009), these results highlight the need to take into account individual differences in reward sensitivity when investigating the effects of conscious and unconscious rewards on executive control.

The current findings echo other studies showing that executive performance fluctuates and can be modified for example by affective stimuli (Dreisbach, 2006; Chiew and Braver, 2014). More specifically, our work is in line with previous research on the influence of motivation showing that rewards can improve executive functioning (Bijleveld et al., 2010; Jimura et al., 2010; Capa et al., 2011, 2013; Bustin et al., 2012; Chiew and Braver, 2014). Here we found that, overall, high rewards (compared to low reward) improved memory-updating. A key finding is that individuals’ reward sensitivity mediated these reward-related effects on memory-updating (at least when reward cues were processed consciously). A straightforward explanation is that in contrast with the high BAS group, individuals with low sensitivity to rewards should experience a weaker incentive state when processing the high rewards, leading to a less improvement in memory-updating relative to the low reward condition. However, why both conscious and unconscious rewards improved memory-updating, but only conscious rewards’ effects depended on individual differences in reward sensitivity is open to discussion.

A relevant framework for understanding conscious and unconscious reward processing and its similar or distinctive effects on performance has been suggested by Bijleveld et al. (2012b). This model may be useful to understand the variability of the effects of conscious and unconscious rewards on memory-updating. Accordingly, people first process rewards in subcortical brain structures, such as the striatum (Pessiglione et al., 2008). This initial processing requires little perceptual input, is not consciously experienced, and can directly facilitate performance by prompting task engagement in the service of reward attainment. However, when supraliminal reward cues are consciously perceived, they may undergo full processing, in which case, the brain structures mobilized (e.g., anterior cingulate cortex, dorsolateral prefrontal cortex, and medial prefrontal cortex), in addition to the structures already engaged by initial reward processing, may involve higher-level cognitive functions, such as strategy (Haber and Knutson, 2009). Thus, full reward processing may lead individuals to consciously choose a strategy. Furthermore, when strategy differences emerge, the effects of conscious and unconscious reward cues may also differ, with conscious reward cues either helping or hindering performance.

A good illustration of hindering performance was observed with the low BAS participants (**Figure 2A**). They performed better for the high reward than the low reward, but only in the subliminal condition. No improvement in performance was observed for a high reward in the supraliminal condition. The amount of money at stake in the present study was probably not challenging enough for the low BAS participants, and conscious reflection on reward probably led them to disengage from the pursuit and attainment of reward. This is consistent with the study of Zedelius et al. (2013) which showed that conscious reflection on a money cue can cause people to disengage from attainment of reward. Participants were invited to perform a working memory task in which money cues (coins) serving as rewards or not were displayed supraliminally or subliminally. High money cues led to improved performance even when the coins did not serve as rewards in the subliminal condition, but not in the supraliminal condition. This suggests that consciousness is crucial in regulating effort mobilization toward money cues. In the medium BAS group (**Figure 2B**), the effect of reward was similar in the subliminal and supraliminal conditions, suggesting that participants in the medium BAS group adopted a similar level of task engagement toward the pursuit and attainment of conscious and unconscious rewards. This participant sample is probably the one most frequently studied, and this result is in keeping with our previous study (Capa et al., 2011), which found a reward-related effect in both subliminal and supraliminal conditions. The high BAS group may be a good illustration of conscious reward helping performance. For participants in the high BAS group (**Figure 2C**), the reward effect was greater in the supraliminal condition than the subliminal condition. Consciously reflecting on reward led the high BAS participants to engage more toward attaining the reward and to perform better. This fits well with previous studies showing that an increase of BAS score induced higher task engagement to earn a conscious reward (Boksem et al., 2006, 2008; Hickey et al., 2010).

The existence of unconscious perception is no longer denied. Rather, the controversy has shifted to the depth with which subliminal stimuli can be processed and the limits to unconscious cognition (van Gaal et al., 2012). These limits are source of variability between conscious and unconscious reward effects on executive functions. The present results highlight the limits to the depth with which unconscious stimuli can be processed. In our study, there was no difference in performance across BAS groups when the possibility of winning a large reward was displayed subliminally, suggesting there was no difference in the strategies adopted by the groups. Differences in performance across BAS groups emerged only when the reward was processed consciously, suggesting that consciousness of reward is crucial to triggering specific strategies. This result lends support to the theoretical framework developed by Bijleveld et al. (2012b) which suggests that full reward processing may cause individuals to choose a strategy consciously.

Individual differences in reward sensitivity, as indexed by the BAS scores used in the present study, have been strongly linked to dopaminergic neurotransmission (Gray, 1989; Tomer et al., 2014). Interestingly, two recent studies have investigated whether individual differences in indirect markers of dopaminergic activity modulate the reward-related effects on performance in a tapping task (Veling and Bijleveld, 2015) and a force task (Pas et al., 2014). In both studies, performance correlated with dopaminergic activity when reward cues were presented subliminally, but not when reward cues were presented supraliminally. These results contrast with our finding that performance was moderated by reward sensitivity only when reward information was processed consciously. One possible explanation of these contrasting findings is that the prior studies and the current work differed in the method employed to define inter-individual differences. The prior studies used neurophysiological or behavioral markers of the dopamine system activity, such as eye blink rate (Pas et al., 2014, Study 1), error-related negativity (Pas et al., 2014, Study 2) and performance in the balloon analog risk task (Veling and Bijleveld, 2015). In contrast, we estimated inter-individual differences using self-report measures. It is possible that the explicit self-report of sensitivity to reward led participants to act accordingly when exposed to supraliminal reward cues, hence putting more effort in the implementation of conscious strategies. However, this explanation cannot fully account for the present results, because one can still expect individuals’ trait sensitivity to reward to modulate the effects of subliminal rewards. Then, it is noteworthy that the studies by Pas et al. (2014) and Veling and Bijleveld (2015) involved a tapping task and a force task, respectively, i.e., tasks that place minimal demand on executive control resources. In contrast, we specifically investigated executive performance in a memory-updating task. Such a focus on executive performance may explain the specific pattern of results we obtained. One might speculate that the processing of conscious reward and/or cost-benefit decisions associated with these rewards (Bijleveld et al., 2010, 2012b), are less resource demanding in individuals with high (vs. low) reward sensitivity. Hence, processing conscious reward would be more beneficial for high-BAS individuals during the performance of a task that requires resource demanding, cognitive control processes. In contrast, the more rudimentary unconscious processing of reward would boost motivation (Bijleveld et al., 2010), hence improving executive performance, irrespective of individuals’ reward sensitivity. This interpretation remains, however, speculative and calls for further research.

Interestingly, it has been suggested recently that working memory operations are subjectively costly and therefore cost-benefit decision making would bias the functioning of working memory (Westbrook and Braver, 2016). Individual differences in reward/punishment responsiveness may imply differences in subjective costliness of executive operations and cost-benefit decisions (Franken and Muris, 2005; Suhr and Tsanadis, 2007). Hence, a promising line of future research is to examine the potential relationship between reward responsiveness and cost-benefit decisions underlying executive functioning, for example by evaluating the combined effects of task difficulty and reward as a function of individuals’ BAS/BIS profiles.

It is worth considering our finding that rewards improved memory-updating in light of recent neurocognitive models. On one hand, research demonstrates that reward processing and motivation are intimately linked to dopamine-related circuitry (Kim et al., 2015; Westbrook and Braver, 2016). On the other hand, neurocognitive approaches of executive control suggest that dopamine activity plays a crucial role in the maintenance/updating of working memory content (Braver, 2012). Interestingly, a recent framework proposed by Westbrook and Braver (2016) integrates these two lines of research and suggests that rewards improve the stability of the content in working memory through modulating of frontal dopamine release (Westbrook and Braver, 2016). Importantly, within this framework, striatal dopamine release mediates the incentive-induced improvement of the updating of working memory. On this basis, one might speculate that the improvement in memory-updating we observed in the high reward condition was related to reward-induced modifications of striatal dopaminergic activity.

The present results thus highlight the intimate link between motivation/reward and executive functioning (Pessoa, 2009; Braver et al., 2010). They further show that this link is shaped by the BAS/BIS profile of individuals. However, these results should be considered in light of some limitations. A potentially important limitation of our study is the small number of participants in each group, which limits the generalizability of the findings. Another limitation is that the different groups differed on the three BAS subscales (Drive, Reward Responsiveness, and Fun Seeking). Although the groups were defined according to the Drive subscale, this prevents us to determine which specific dimension of the BAS was responsible for the observed effects. However, it is worth to note that the participants were matched on the BIS component and that the stability of the BAS scores was verified with two administrations, which are strong points of the present research.

## Conclusion

We identified different behavioral responses to consciously vs. unconsciously processed reward stimuli and have shown that individual differences in reward sensitivity are a key factor for explaining variations in executive performance aimed at reward attainment. We found that the modulatory role of reward strengthened with the increase in the BAS scores, but only when the reward was processed consciously. This result suggests conscious processing of reward is crucial for the existence of specific strategies pertaining to reward sensitivity in tasks requiring executive control.

## Ethics Statement

This study was carried out in accordance with the recommendations of the centre of human investigations of Strasbourg with written informed consent from all subjects. All subjects gave written informed consent in accordance with the Declaration of Helsinki. The protocol was approved by the centre of human investigations of Strasbourg. All procedures performed were in accordance with the ethical standards of the institutional and/or national research committee and with the 1964 Helsinki declaration and its later amendments or comparable ethical standards.

## Author Contributions

All authors listed have made a substantial, direct and intellectual contribution to the work, and approved it for publication.

## Conflict of Interest Statement

The authors declare that the research was conducted in the absence of any commercial or financial relationships that could be construed as a potential conflict of interest.

## Footnotes

1 Each of the three BAS subscales can potentially predict reward-related effects (Hickey et al., 2010; Braem et al., 2012). Nevertheless, we choose to create the groups of participants on the basis of the BAS Drive subscale, because it has been found to show the highest construct validity, and it has been suggested to be the best predictor of reward-induced behavior (Carver and White, 1994; Hickey et al., 2010; see also Ross et al., 2002).

2 These distribution parameters were obtained from our data instead of the original Carver and White (1994) distribution. This was done because we used the French version of the BIS/BAS scales and little is currently known about the distribution of scores on this French version.

3 This prime duration was selected to approximate the 27 ms prime duration in Capa et al. (2011) given our monitor refresh rate (85 Hz).
